# Supplementary material for: High infectiousness immediately before COVID-19 symptom onset highlights the importance of continued contact tracing
Source: eLife. 2021 Apr 26;10:e65534. doi: 10.7554/eLife.65534 (PMC8195606; doi:10.7554/eLife.65534)
Supplement: Supplementary file 1. — Point estimates (obtained by calculating the posterior mean of the vector of fitted parameters, θ, as described in Materials and methods) and 95% credible intervals for fitted parameters are given for each model. Note that the parameters μF and αF in the Ferretti model do not have the same epidemiological interpretations as the parameters μ and α in our mechanistic approach. [file elife-65534-supp1.docx]

| **Model** | **Parameter** | **Point estimate** | **95% credible interval** |
| --- | --- | --- | --- |
| Variable infectiousness | Shape parameter of latent period, $k_{E}$ | 3.38 | 2.45-4.22 |
|  | Reciprocal of mean symptomatic infectious period, $\mu$ | 0.37 day^-1^ | 0.26-0.52 day^-1^ |
|  | Ratio between transmission rates in the presymptomatic infectious and symptomatic infectious stages, $\alpha$ | 2.29 | 0.88-6.15 |
| Constant infectiousness | Shape parameter of latent period, $k_{E}$ | 2.80 | 2.10-3.49 |
|  | Reciprocal of mean symptomatic infectious period, $\mu$ | 0.46 day^-1^ | 0.36-0.58 day^-1^ |
| Ferretti | $\mu_{F}$ | −5.45 days | (−10.71)-(−0.60) days |
|  | $\sigma_{F}$ | 1.84 days | 1.43-2.25 days |
|  | $\alpha_{F}$ | 11.04 | 1.22-113.29 |
| Independent transmission | Mean generation time, $m_{gen}$ | 5.57 days | 5.08-6.09 days |
| and symptoms | Standard deviation of generation times, $s_{gen}$ | 2.32 days | 1.83-2.91 days |
